# Chemical Composition, Nutritional Value, and Acceptance of Nut Bars with the Addition of Edible Insect Powder

**DOI:** 10.3390/molecules27238472

**Published:** 2022-12-02

**Authors:** Stanisław Kowalski, Joanna Oracz, Magdalena Skotnicka, Anna Mikulec, Dorota Gumul, Barbara Mickowska, Aleksandra Mazurek, Renata Sabat, Anna Wywrocka-Gurgul, Dorota Żyżelewicz

**Affiliations:** 1 Department of Carbohydrate Technology and Cereal Processing, Faculty of Food Technology, University of Agriculture in Krakow, 122 Balicka Street, 30-149 Krakow, Poland; 2 Institute of Food Technology and Analysis, Faculty of Biotechnology and Food Sciences, Lodz University of Technology, 2/22 Stefanowskiego Street, 90-537 Lodz, Poland; 3 Department of Commodity Science, Faculty of Health Sciences, Medical University of Gdansk, 80-210 Gdansk, Poland; 4 Department of Engineering Sciences, Academy of Applied Science in Nowy Sacz, 1a Zamenhofa Street, 33-300 Nowy Sacz, Poland; 5 Department of Plant Product Technology and Nutrition Hygiene, Faculty of Food Technology, University of Agriculture in Krakow, 122 Balicka Street, 30-149 Krakow, Poland

**Keywords:** edible insects, amino acid profile, nutritional value of protein, fatty acid profile, sensory analysis

## Abstract

Six types of nut-based bars with the addition of edible insect flour were obtained. Flours made from three different insects (*Tenebrio molitor* L., *Acheta domesticus* L., *Alphitobius diaperinus* P.) were used at two different additive levels (15% and 30%) in relation to the weight of the nuts. The addition of insect flour significantly increased protein content and the insoluble fraction of dietary fiber. The largest amount of these compounds was found in bars with 30% cricket flour, 15.51 g/100 g and 6.04 g/100 g, respectively, in comparison to standard bars, 10.78 g/100 g and 3.14 g/100 g, respectively. The greatest consumer acceptance was found in relation to bars with buffalo worm flour. The overall acceptance of these bars was 6.26–6.28 points compared to 6.48 for standard bars. Bars and raw materials were characterized by the high biological value of the protein. Cis linoleic acid dominated among unsaturated fatty acids. The percentage of this compound was in the range of 69.56%, for bars with a 30% addition of buffalo worm flour, to 73.88%, for bars with 15% cricket flour. Instrumental analysis of taste and smell compounds showed the presence of compounds such as 3-methylbutanoic acid, hexanal, and 2,3-pentanedione.

## 1. Introduction

In recent years, the world has faced a series of new challenges, of which the issue of feeding humanity appears to be of the utmost importance. Global population growth is projected to reach 10 billion by 2050, posing enormous challenges to all sectors involved in food production. Classic animal production that provides humans with the main amount of protein containing the necessary amino acids may turn out to be insufficient. Breeding of cattle, pigs, and poultry requires a lot of energy, requires space, and is both water- and feed-intensive. To this should be added other environmental issues, such as greenhouse gas emissions [1]. Climate change and food waste are other factors contributing to food decline worldwide [1,2,3]. Edible insects are one of the proposed sources of protein to overcome deficiencies [4]. Insect powders, also called insect flours, are used among other applications for bread formulations [5,6,7], which, due to the high protein content characteristic for this type of product, contributes to its increased nutritional value. Edible insects are part of the diet of about 2 billion people worldwide, especially in China, Thailand, Vietnam, central and south Africa, Mexico and Brazil, and according to the Food and Agriculture Organization (FAO), about 1900 insect species are consumed as food products [8]. These data do not apply to the so-called Western societies, where entomophagy is on a marginal level and insect consumption is considered to be disgusting. The resistance and reluctance of Western societies to consume edible insects is related to their conservatism and neophobia [4,9]. Several efforts, supported by the FAO, are being made to increase the acceptance of products enriched with edible insects [8]. The acceptance of such products increases when insects are in a form that is invisible to the consumer, i.e., as powders, extracts, or concentrates [9,10]. It was found that addition of cinereous cockroach flour can increase the proportion of protein in bread by over 130% [11]. In world markets, including European markets, there is a whole range of commercial products containing powder from whole edible insects or their protein preparations. This group includes bread, snacks, meatballs, cookies, chocolate bars, and even beer (Table S1).

Among the most frequently and willingly used edible insects in the creation of food products are yellow mealworm (*Tenebrio molitor* L.) and buffalo worm (*Alphitobius diaperinus* P.) at their larval stage, as well as house cricket (*Acheta domestica* L.). These insects are used to enrich, inter alia, products such as bread [5,6,12,13,14], sponge cakes [15], and snacks [16], or are used as a meat replacement in hamburger formulation [9]. Dried insect powder can contain up to 65% protein (usually about 50%), 28% fat (with predominant C16:0 and C18:2 (cis-9,12) fatty acid fractions), and minor components such as phosphor, potassium, and vitamin C [5,17,18,19].

The size of the global snacks market was valued at USD 1450.4 billion in 2021 and is projected to grow at a compound annual growth rate (CAGR) of 2.7% between 2022 and 2030. The changing lifestyle seen mainly among Americans and Europeans, involving the consumption of smaller meals, contributes to the increased consumption of snacks. That is why bars, especially those which are a healthier form of snack product, are in growing demand. Changing consumer behavior, giving up traditional animal protein, is mainly due to people’s growing concerns about animal welfare or sustainable development. Consumers are increasingly looking for products that will help them maintain a healthy and balanced diet. Functional snacks are becoming more and more popular, and the expectations and needs of young customers are evolving at a rapid pace [20].

Nut bars are a type of food aimed at young, active people who want to quickly supplement the required food ingredients. These people are often more open to nutritional information and, at the same time, are highly aware of the ethical and environmental risks associated with food production. It is extremely important that, when facing the threat of failures in traditional food production, necessary alternatives to meet the nutritional needs of humanity are found. Promoting entomophagy among such people may contribute to increasing interest in eating edible insects in Western societies, or at least reducing the reluctance to introduce new food products to the market. This can be achieved by introducing products dedicated to young generations for whom they will soon become a natural alternative and nutritional choice. For this reason, it was decided that this study would focus on the development of nut-based bars enriched with powders from various edible insects. The most popular and consumer-accessible preparations of edible insects were used, i.e., yellow mealworm (*T. molitor*), buffalo worm (*A. diaperinus*), and house cricket (*A. domesticus*) powders.

## 2. Results and Discussion

### 2.1. Chemical Composition of the Bars

Among the insect flours, cricket flour was characterized by significantly higher protein content (55.18), fat (29.01), chitin (9.92), and insoluble fractions of dietary fiber (18.48 g/100 g) compared to the others (Table 1). Nuts, especially hazelnuts, turned out to be the source of the soluble dietary fiber fraction in the bars. In comparison to standard bars, the addition of insect flour significantly increased protein content from 13.11 (TM15) to 15.51 g/100 g (CF30), the insoluble fraction of dietary fiber from 4.14 (BW15 and TM15) to 6.04 g/100 g (CF30), and chitin from 0.77 (BW15 and TM15) to 2.35 g/100 g (CF30) (Table 1). These results are consistent with the data presented by other authors [5,6,13,19]. Rumpold and Schlüter [21,22] also considered edible insects as a food source of high nutritional value, due to their high protein, fat, vitamin, and mineral content.

Chitin is a type of carbohydrate polymer that forms the exoskeleton of most arthropods, including insects. Selenius et al. [23] demonstrated the prebiotic nature of chitin, due to its effect on the improvement of intestinal microbiota. They observed its effect on the proliferation of naturally occurring microflora in the gut. It has been suggested that chitin, as a prebiotic, indirectly helps to prevent diseases transmitted by microorganisms present in food and facilitates the digestion of food. Their research also showed chitin had an effect on improvement of the growth of *Lactobacillus rhamnosus* GG and the inhibition of *Escherichia coli* TG [23]. Fernandes et al. [24] reported that chitin and its derivatives influence the multiplication and development of beneficial species of intestinal bacteria such as *Bifidobacteria* and *Lactobacillus*.

### 2.2. Protein and Amino Acid Composition

On the basis of electrophoretic profiles of raw materials, it can be concluded that the molecular weight of hazelnut proteins was in the range of about 8–70 kDa, with the main protein fractions approximately 17–20 kDa (two protein bands) and 32–37 kDa (three bands) (Figure S1). Cashew protein bands were in the range of 10–100 kDa, but the majority of proteins were grouped in two fractions with molecular weights of approximately 17–23 kDa and 30–32 kDa. There was also a peptide fraction with a molecular weight of about 10 kDa. Cricket flour’s main protein bands were concentrated in two ranges of molecular weight: 13–20 kDa and 25–40 kDa. Additionally, several slight bands also appeared in a range of higher molecular weights up to 200 kDa (Figure S1). The molecular weight of mealworm flour proteins was in the range of about 12–60 kDa, with dominating bands around 58, 50, 37, 25–27, and 12 kDa. Buffalo worm flour protein bands were present in a wide range of molecular weights (from 12 to 200 kDa), with distinctive bands at 12, 27, 37–39, 60, and 70 kDa (Figure S1).

In the amino acid profile of both raw materials and the obtained bars, endogenous amino acids dominated; the highest sum of their content was characteristic for the standard bar, and the lowest for hazelnuts (821.95 and 615.59 mg/g of protein, respectively). Most bars with insect flour did not differ significantly in terms of the sum of endogenous amino acids (Table S2). The standard bar (519.88 mg/g of protein) had the highest content of exogenous amino acids (Table 2). Among the bars with insect flours, apart from CF30, no significant differences in the total amount of exogenous amino acids were observed. Both the combination of cashew and hazelnuts and the addition of insect flour contributed to an increase in the nutritional value of protein (Table 3). Insect flour and the obtained bars were characterized by a high biological value in terms of protein. The AAS value did not drop below 100% for any of the amino acids (Table 3). Cricket flour was characterized by significantly higher protein content (55.18%) compared to other raw materials, which ranged from 16.64% for hazelnuts to 49.51% for buffalo worm flour (Table 2). These values are in line with results previously published by other authors [5,6,13,15,25]. Other authors also reported similar amino acid content in edible insects [6,15,17,21,22].

Therefore, the content of amino acids in the studied bars was compared to the model proposed by the FAO/WHO [26]. Based on the amino acid score (AAS), an indicator of protein quality in diets, it was calculated that the amino acids that limited the biological value of the protein of the tested nuts were leucine and lysine (Table 3). The addition of insect flour increased the biological value of the protein in the obtained bars. Due to the high content and well-balanced level of exogenous amino acids, insect protein bars have high biological value (Table 3).

### 2.3. Fatty Acid Composition

The fatty acid profile of the bars was correlated with the fatty acid profile of the raw materials used for their production. Particular insect flours and nuts differed significantly in the content of individual fatty acids. Their profile was dominated by cis-oleic acids (C18:1(9) n-9) (from 82.44 for hazelnuts to 73.87% for bars with 30% addition of cricket flour) (Table 4). The content of this acid was also dominant in the bars. Significantly the lowest content was found in bars with 30% addition of buffalo worm flour (69.56%, and significantly the highest with 15% and 30% addition of cricket flour (73.88% and 73.87% respectively) (Table 4). C18:2 (9.12) n-6 cis linoleic acid dominated among unsaturated fatty acids, with the highest level in standard bars (12.75%) and the lowest in BW30 bars (10.93%).

The obtained results, regarding fatty acid profile of insect flour, are consistent with the data presented by other authors [4,6,15,17,25]. For the obtained fatty acid profile of edible insects, the polyunsaturated to saturated fatty acid (PUFA/SFA) ratio was calculated. This index allows assessment of the nutritional quality of foods based on their fatty acid compositions. From a nutritional point of view, the PUFA/SFA ratio is unfavorable, ranging from 0.66 for BW30 bars to 0.91 for CF30 bars (Table 4). In judging the nutritional value of edible insect fat, it should be borne in mind that the views on the harmful effects of saturated fatty acids have recently been revised [27].

### 2.4. Consumer Acceptance Analysis

After assessing the acceptance of six samples against the control sample (Figure S2), it was found that the highest overall acceptance of the bars was demonstrated by samples without the addition of insect flour ST-6.48 (Table 5). However, bars with the addition of buffalo worm flour were rated only slightly lower, both in the case of 15% buffalo worm flour (BW15), with acceptance of 6.26, and 30% buffalo worm flour (BW30), with acceptance of 6.28 (Table 5). It seems that increasing the proportion of buffalo worm flour had no effect on the overall acceptance rating. The lowest overall acceptance score was obtained for bars with mealworm flour. Among the analyzed sensory features, the smallest differences in results were obtained for assessments of appearance and color. In fact, the appearance and color of bars with the addition of 30% buffalo worm flour and 30% cricket flour were rated the lowest, which, however, was not reflected in the overall acceptance rating, especially in the case of buffalo worm, where this variant was rated the highest (Table 5). The decisive descriptor determining final acceptance was likely the taste of the bars tested. Texture is also an important element of the sensory evaluation. In the context of texture, the form of the insect from which the flour is made, and its amount, are of key importance. The more insect flour added, the lower the texture score. Only with cricket flour was the variable addition of flour irrelevant. The structure of bars including cricket in their composition was rated the lowest. This is probably related to the fact that only in this one variant was the adult form, the imago, used. These samples contained more chitin, which is an important factor determining texture. This is important information for manufacturers designing novel functional food based on edible insects.

**Figure 1:**
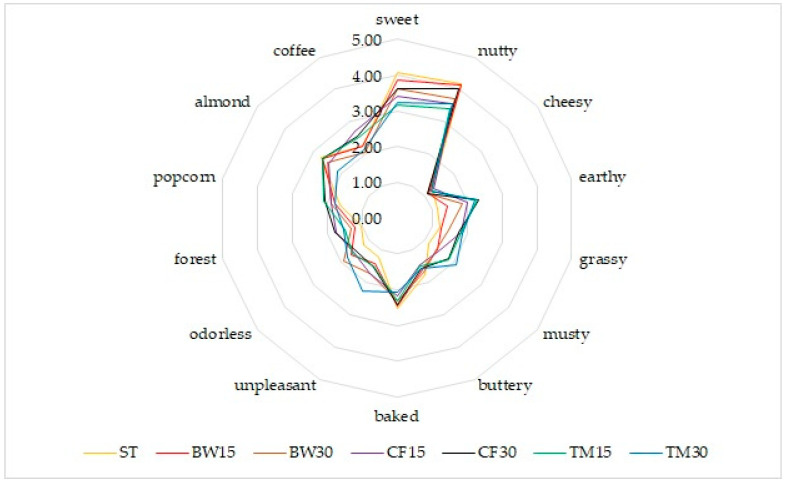
Organoleptic characteristics of bars—smell profile.

On the basis of a detailed analysis, the most frequently declared features of smell, taste, and texture were selected. Odor analysis showed that popcorn, coffee, and almond were the most frequently identified odors among all samples (Figure 1). However, it was the latter that was felt most strongly. Similar results were obtained for the control sample, which means that enriching the products with insect flour does not significantly change the smell of the product. On the other hand, among the undesirable odors, earthy and grassy smells appeared only sporadically (Figure 1). On the other hand, it is worth noting that for bars with mealworm, a much higher percentage than in the case of other samples indicated an unpleasant smell that is difficult to identify. It is worth emphasizing that the performed instrumental analysis explains, to some extent, the observed organoleptic dependencies (higher content of 3-methylobutanoic acid, hexanal, and 2,3-pentanedione in mealworm flour-based bars compared to other bar samples).

**Figure 2:**
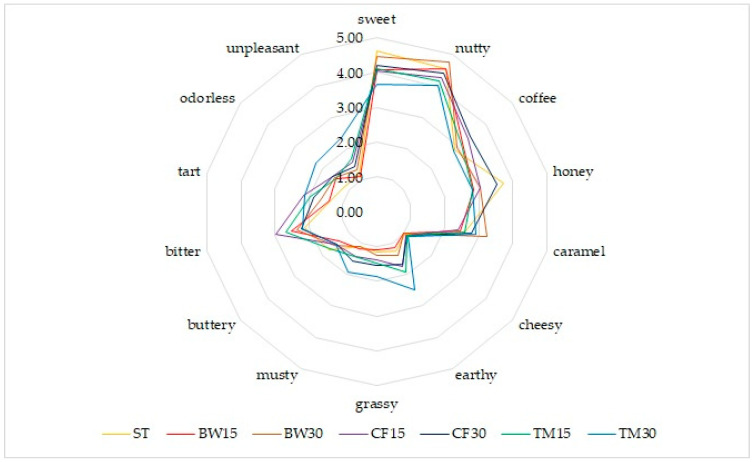
Organoleptic characteristics of bars—taste profile.

A nutty flavor was most pronounced in the case of bars with 15% and 30% buffalo worm content compared to the control sample (Figure 2). For the remaining variants of bars with insects, a lower frequency of perceiving a nutty flavor was noted. In both variants of the cricket bars, the largest increase in the frequency of perceiving a coffee flavor was observed. An earthy taste was reported for bars with the addition of mealworm, which was directly proportional to insect content.

An unpleasant taste was most often reported for the variant with 30% mealworm content, as demonstrated by odor identification.

**Figure 3:**
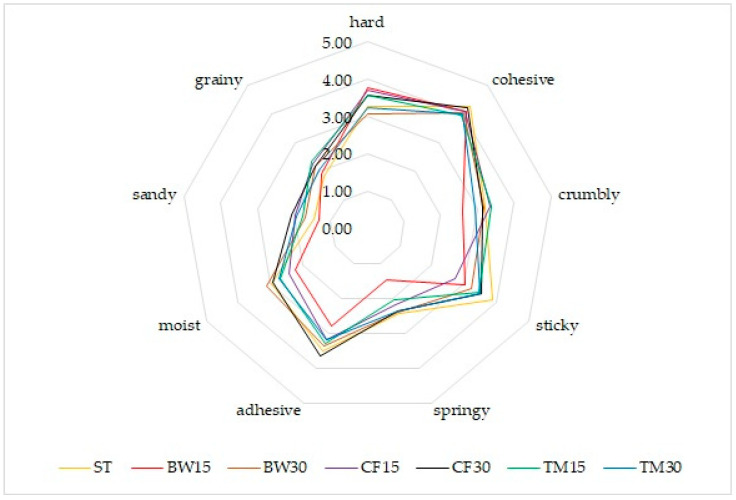
Organoleptic characteristics of bars—texture profile.

Among the control bar texture descriptors, the most frequently reported texture characteristics were the firm and cohesive texture expected for this type of food product. With the addition of 15% insect flour, the identified structure was hard and cohesive, but the addition of a larger amount of insect flour, regardless of the type, resulted in deterioration of the texture (Figure 3).

### 2.5. Instrumental Analysis of Sensory Features

**Figure 4:**
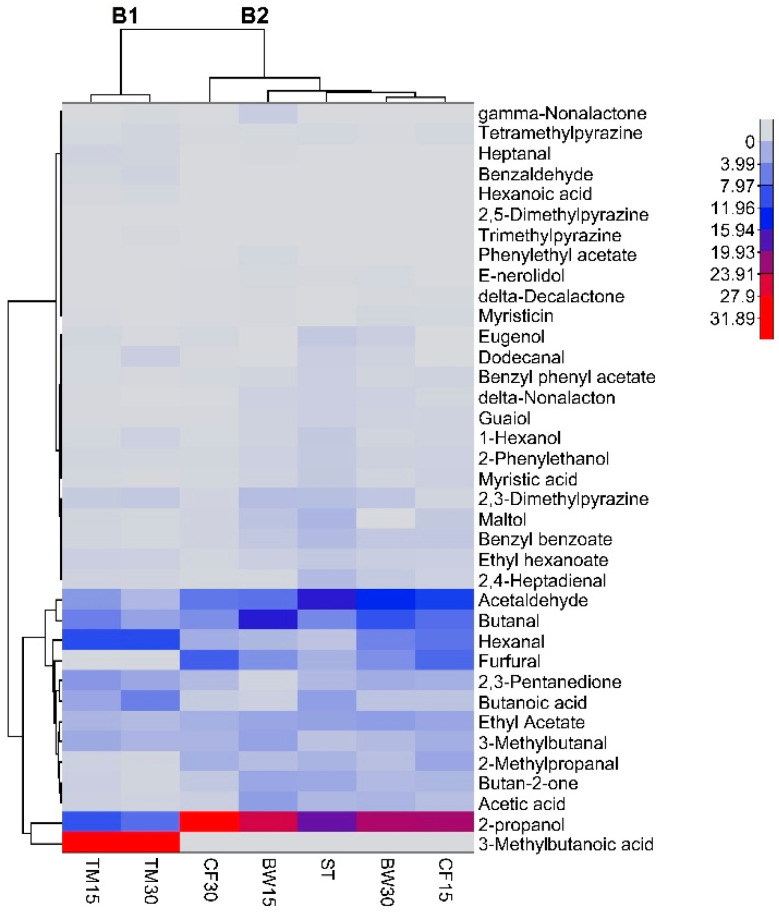
Heatmap showing the distribution and concentration of individual volatile compounds in bars.

Results of the analysis of aroma compounds in the control and edible insect flour-substituted bars analyzed by an electronic nose are presented as a heatmap (Figure 4). A total of 38 volatile compounds were identified in the analyzed bars, including aldehydes, ketones, alcohols, esters, pyrazines, lactones, phenols, acids, and pyranones. The results showed that the control bars and those substituted with insect flours differed significantly in terms of the content of aroma compounds. An agglomerative hierarchical cluster analysis coupled to the heatmap was generated to depict the relationship between insect flour supplementation and the concentration of individual volatile compounds in bars (Figure 4). Euclidean distance was used as the distance measure for determining the similarity between samples, and the axis of the map showed the samples and volatile compounds. Different colors in the rectangles indicate the concentration of total amounts of different classes of aroma compounds found in the samples. The results showed that the profile of volatile compounds for the bars depends on the type and proportion of insect flours used. In the heat map, two main clusters in columns were generated and highlighted by hierarchical clustering (B1 and B2). The different clusters of bar samples indicate significant differences in aroma compound profiles. It can be observed that bars with mealworm flour can be grouped into the first cluster (B1), which obviously differed from the other samples. According to hierarchical cluster analysis in the heat map, the type and concentration of volatile compounds in the control bars show some similarity to the amounts of these compounds in the samples supplemented with buffalo worm and cricket flours, regardless of their percentage amount. Thus, these samples were grouped as the second cluster (B2). The individual flavor compounds were also grouped into two main clusters in the dendrogram and were further grouped into different subclusters according to the similarity of their concentration patterns in the analyzed bar samples. As can be seen in Figure 4, the most abundant flavor and aroma compounds in the control bars and those supplemented with buffalo worm and cricket flours were acetaldehyde and 2-propanol, which are responsible for pungent choking and sharp musty alcoholic odors, respectively [28]. Samples grouped in the second cluster also contain significantly higher amounts of furfural, acetaldehyde, 2-propanol, and 2-methylpropanal than those from the first cluster. Bars supplemented with mealworm flour, regardless of the percentage amount, were characterized by significantly higher 3-methylbutanoic acid, hexanal, and 2,3-pentanedione content than other bar samples. These compounds provide pungent, acid, cheese, sweet, creamy, and nutty odors.

The results showed that the proportions of different insect flours contributed to notable differences in the concentrations of volatile compounds. In addition, it was observed that bars enriched with insect flours, regardless of the type of flour, had significantly higher 3-methylbutanal content than the control samples, which is responsible for pleasant caramel, fruity, and malty notes [29]. The Maillard reactions, caramelization, and lipid oxidation reactions play a significant role in shaping the flavor of roasted nuts. During these reactions, some heterocyclic volatile compounds, such as furans, ketones, pyrazines, pyrroles, aldehydes, and pyridines, are formed in nuts [30]. Thus, some specific compounds related to this reaction in nuts, such as dimethylpyrazines, 2,4-heptadienal, 2-phenylethanol, phenylethyl acetate, and maltol, were present in significantly higher amounts in control samples compared to those enriched with insect flours. These findings indicate that the type of insect flour used and the proportion of each flour in the recipe had a significant impact on the profile and concentration of volatile compounds. The increased amount of 3-methylobutanoic acid with increasing substitution of mealworm flour is due to the higher initial content of this acid in this additive (Table S3). In addition, the higher fat content in this flour, compared to buffalo worm and cricket flours [6], may be responsible for the generation of higher amounts of hexanal via lipid oxidation [28,29]. On the other hand, butanal and furfural, which give products a chocolate and caramel taste, were present in significantly higher amounts in the bars enriched with buffalo worm and cricket flours compared to other samples. Therefore, an increase in the proportion of insect flours during the production of bars led to significant differences between volatile compounds due to their high protein and fat content and chemical reactions such as Maillard reactions, caramelization, and lipid oxidation.

**Figure 5:**
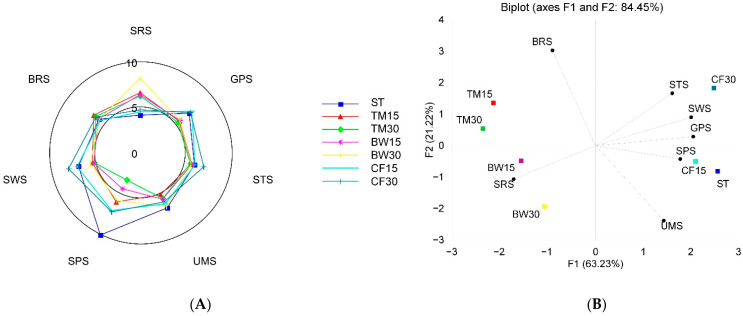
Radar chart representing the taste scores of bars (**A**) and a PCA graph showing the relationship among samples and tastes. SRS—sourness, GPS—metallic, STS—saltiness, SPS—spiciness, UMS—umami, SWS—sweetness), and BRS—bitterness (**B**).

Taste quality (distinguished between different tastes) and its intensity in water extracts made from the control and edible insect flour-substituted bars were evaluated using an electronic tongue with seven cross-selectivity sensors. The results showed that enrichment of bars with insect flour significantly affected the taste attributes of the studied samples (Figure 5A,B). The bars with insect flours showed a significantly stronger sour taste than the control samples, regardless of the type and amount of insect flour. The greatest increase in the sourness of bars was observed with 30% buffalo flour. However, the intensity of the other tastes depended largely on the type of insect from which the flour was obtained. The addition of cricket flour led to a significant increase in the intensity of sweet and salty tastes compared to control and other enriched samples. Increasing the amount of this insect flour increased the intensity of these tastes. The highest scores for bitter taste were given to bars with a 15% and 30% share of mealworm flour. On the other hand, enriching the bars with insect flour did not increase the intensity of the umami taste. Principal component analysis (PCA) was used to visualize the relationship between the samples and variables (taste scores) in order to establish their differences and similarities. Figure 5B shows that 84.45% of the total variance in the data is represented by PC1 and PC2. Of these two principal components, PC1 described 63.23% of the total variation, and PC2 explained 21.22% of the variation. In the biplot, it can be seen that the analyzed bars and their tastes formed four separate clusters. The first cluster included the control bars and bars with a 15% share of cricket flour, which had noticeably less variation in taste profile and the lowest sour taste intensity.

The second cluster only contained the bar with 30% cricket flour, which is characterized by the sweetest taste among the samples tested. The third cluster includes bars with mealworm flour. These samples are characterized as more bitter than other bars. The fourth cluster contained bars with buffalo worm flour, which are characterized by a pronounced sour taste. The observed relationship between increases in sourness, sweetness, bitterness, and saltiness and the fortification of bars with insect flour may be related to their high protein, mineral, and organic acid content [6,31]. Therefore, the results of the current study indicate that the amount and type of ingredients used in the production of bars may affect their organoleptic properties.

## 3. Materials and Methods

### 3.1. Materials

Nut bars were made of hazelnuts (Bakalland Ltd., Warsaw, Poland) and cashew nuts (Bakalland Ltd., Warsaw, Poland), in a 1:1 ratio, and honeydew honey (Huzar Ltd., Nowy Sacz, Poland), in an amount equal to 0.75% of the weight of the nuts. In the enriched bars, 15% and 30% insect flour from mealworm *T. molitor* (DeliBugs, Lelystad, The Netherlands), buffalo worm *A. diaperinus* (DeliBugs, Lelystad, The Netherlands), or cricket *A. domesticus* (DeliBugs, Lelystad, The Netherlands) was added based on the weight of the nuts.

#### Preparation of Nut Bars with Insect Powder

Nuts were roasted in a pan at 180 °C for 5 min. Crushed nuts for standard bars and crushed nuts and insect flour for the remaining bars were combined with dissolved honey (35 °C), thoroughly mixed, placed in silicone molds with dimensions of 75 × 30 × 30 mm, and placed in a refrigerator at 4 °C for 120 min.

### 3.2. Methods

#### 3.2.1. Chemical Analysis

Basic physicochemical analysis of standard bars, as well as bars with the addition of edible insect powder (flour), was conducted according to AOAC methods [32]. Chitin content was determined according to the colorimetric method [33]. Analyses were performed in triplicate.

#### 3.2.2. Amino Acid Analysis

Analysis was based on the methods of Moore and Stein [34], Davidson [35], Smith [36], and Moore et al. [37]. Freeze-dried samples were hydrolyzed using 6 M hydrochloric acid (with 0.5% phenol) at 110 °C for 24 h under an inert gas atmosphere. After the hydrolysis was completed, samples were lyophilized, dissolved in loading buffer (sodium citrate buffer pH 2.2), and filtered through 0.45 μm syringe filters. Amino acids were determined using an automatic liquid chromatograph with post-column derivatization and UV detection (AAA400, Ingos, Czech Republic). A standard amino acid solution (Sigma, St. Louis, MO, USA) was used for calibration. Components of hydrolysates were separated on a strong cation ion-exchange resin, followed by post-column derivatization with a ninhydrin reagent and photometric detection at wavelengths of 570 and 440 nm. Methionine and cysteine were analyzed as stable derivatives after performic acid oxidation followed by a standard liquid-phase hydrolysis protocol. Quantification of the acquired data was computed using the chromatographic device’s software (Chromulan, Pikron, Czech Republic). Since tryptophan degrades during acid hydrolysis, it was not determined. Asparagine and glutamine turn to aspartic and glutamic acid and were estimated in these forms. Analysis of each sample was performed in four repetitions.

To express the nutritional value of the protein, amino acid score (AAS) was calculated according to the FAO/WHO (1991) (Equation (1)):

AAS = (mg of amino acid in 1 g of test protein)/(mg of amino acid in reference pattern*) × 100(1)

* Recommended amino acid scoring patterns for adolescents and adults according to the FAO [38].

#### 3.2.3. SDS-PAGE Electrophoresis

Extraction was performed using a mixture containing 125 mM Tris-HCl pH 6.8, 4% SDS, 20% *v/v* glycerol, and DTT 50 mg/mL at 100 °C for 5 min. The Schägger-von Jagow method [39] with protein molecular weight standards in the range of 10–180 kDa (PageRuler Prestained Protein Ladder, Thermo Scientific, Waltham, MA, USA) was used to perform SDS PAGE electrophoresis. Gels were run in Mini Protean Tetra Cell electrophoresis equipment (Bio-Rad, Hercules, CA, USA) and stained with Coomassie Brilliant Blue R-250.

#### 3.2.4. Determination of Fatty Acid Profile

Fatty acid profile was determined based on the AOAC method [32]. The extracted fat (15 mg) was saponified using 0.5 mL of 0.5 N KOH (methanol solution) at 85 °C. Subsequently, 1 mL of derivatizing solution (12% BF_3_ (methanol solution)) was added and heating was continued at 85 °C. To isolate the fatty acid methanol esters, 1 mL of hexane and 5 mL of saturated NaCl solution was added to the sample. Chromatographic separation was obtained using a Shimadzu GC2010Plus Chromatograph (Shimadzu Corp., Kyoto, Japan) with a flame ionization detector (FID). Operating parameters were as follows: FID detector temp., 250 °C; temperature dispenser, 220 °C; oven temp, 80 °C (held for 2.5 min), heated to 160 °C (60 °C/min) and held for 35 min; heated to 230 °C (15 °C/min) and held for 5 min; heated to 240 °C (5 °C/min) and held for 5.5 min. An SH-FAME column (30 m × 0.32 mm × 0.25 μm) was used and the carrier gas was helium (1.6 mL/min). Split ratio 100. Identification of individual fatty acids in the form of their methyl esters was carried out by comparing the retention times of the peaks of the standard substances (Sigma-Aldrich Co., St. Louis, MO, USA) and the peaks of the analyzed samples. Analyses was performed in four repetitions.

#### 3.2.5. Consumer Acceptance Analysis

Organoleptic analysis of the obtained bread was carried out by 36 panelists aged 20 to 29 who were selected randomly from students at the Medical University of Gdansk on the basis of a descriptive analysis test. During the planning stage of the experiment, the sample size was selected at a level generating statistical conclusions with appropriate accuracy and certainty, and the probability of the test detecting effects of a given size was examined based on test power analysis and interval estimation of the sample size. The performed statistical analysis showed that all the tests that were made for the acceptance assessment of the seven test samples of bars can be considered representative. Based on collected data from the assessment of acceptance level, the minimum sample size for each bar should be at least 24 for each variant for all measurements. The evaluators provided answers on the previously prepared evaluation cards. Each assessor received his own set of samples (six samples + control) in a random order. A seven-point Likert scale with appropriately selected descriptors was used for the evaluation: appearance, smell, texture, taste, and the overall acceptability of the bread were rated on a scale from one to seven (seven—extremely acceptable, one—not at all acceptable). Additionally, the flavor, aroma, and texture profile of the bars obtained were determined. In the case of smell, the assessors had a choice of the eleven most frequently reported smells: eight positive smells (sweet, nutty, buttery, popcorn, almond, coffee, cheese, grain) and three negative smells (earthy, grassy, musty), with one identified as unpleasant. Regarding the taste assessment, nine basic tastes were identified: five desirable tastes (sweet, nutty, buttery, cereal, and coffee) and four undesirable tastes (musty, bitter, tart, and odorless), with one identified as unpleasant. Regarding the texture assessment, eight differentiators characterizing the texture and consistency of bars were identified: hard, crumbly, sticky, springy, adhesive, sandy, and grainy.

Analysis of the three basic discriminants was based on the assessors selecting one answer out of five possible: one—completely imperceptible; two—almost imperceptible; three—hard to define; four—slightly perceptible; and five—completely perceptible.

The evaluators were healthy, took no medications or supplements, and followed no special diets. All participants voluntarily signed consent forms in order to participate in the research, which was approved by the Independent Bioethical Committee for Scientific Research at the Medical University of Gdansk (NKBBN/346/2021). This study complies with the ethical principles of non-harm, charity, justice, and autonomy as enshrined in the ethical provisions of the revised 2013 Declaration of Helsinki. Before the study, all evaluators completed the Food Neophobia Study (FNS) questionnaire. Only those who did not show nutritional neophobia participated in the study.

#### 3.2.6. Analysis of Volatile Compounds Using an Electronic Nose

Electronic nose (E-nose) analysis of volatile flavor compounds was carried out according to the method of Żyżelewicz et al. [40], with some modifications. The E-nose analyses were performed using a commercial Heracles II electronic nose (Alpha MOS, Toulouse, France) equipped with an HS-100 autosampler, a sensor array unit, two flame ionization detectors, and two columns working in parallel mode: a non-polar column (MXT5: 5% diphenyl, 95% methylpolysiloxane, 10 m length, and 180 µm diameter) and a slightly polar column (MXT1701: 14% cyanopropylphenyl, 86% methylpolysiloxane, 10 m length, and 180 µm diameter). Samples of 1.0 g were weighed into 20 mL screwcaps sealed with a magnetic cap with PTFE-silicone septa, placed in the autosampler, and incubated for 20 min at 50 °C with shaking at 500 rpm. Subsequently, 1000 µL of the headspace phase was collected and injected into a gas chromatograph. The temperature program started at a temperature of 50 °C (held for 5 s), which was increased to 260 °C at 3 °C/s and held for 35 s. The total separation time was 110 s. Calibration of the apparatus was carried out using a solution of alkanes (from n-hexane (C6) to n-hexadecane (C16)). The retention times of n-alkanes were used to determine the Kovats indices and identify the volatile compounds using AromaChemBase software (Alpha MOS, Toulouse, France). Each sample was measured in triplicate. Instrument control, data acquisition, and evaluation were conducted with Alphasoft 14.2 and AroChembase (Alpha MOS, Toulouse, France) software. Principal component analysis (PCA) was performed using Alphasoft software (Alpha MOS, Toulouse, France) to determine the dissimilarities among the samples in terms of volatile components.

#### 3.2.7. Analysis of Chemical Compounds Using an Electronic Tongue

Instrumental taste analysis of samples was carried out using the Alpha MOS ASTREE II electronic tongue (e-tongue) instrument (Alpha MOS, Toulouse, France), which consists of a 48-position autosampler for automated analysis of a sample set in reproducible conditions (time, stirring), an array of liquid sensors (set #5), and a reference electrode (Ag/AgCl). The sensor set #5 array used in this study contains seven sensors (SRS—sourness, GPS—metallic, STS—saltiness, SPS—spiciness, UMS—umami, SWS—sweetness, and BRS—bitterness) and was designed for food and beverage applications [41]. For e-tongue analysis, 1.0 g of each sample was extracted with 50 mL of distilled water using a water bath with shaking for 20 min at 60 °C, and the extracts were centrifuged at 4800× *g* for 10 min at 20 °C. The supernatants were filtered through Whatman no. 4 filter paper and were transferred to the glass containers of the 25 mL autosampler. Analysis time was set at 120 s at 25 °C and data from the last 30 s were used for acquisition. Between measurements, the sensors were rinsed with distilled water for 120 s. Prior to analysis, the e-tongue sensors were conditioned (0.01 mol/L HCl), calibrated (0.01 mol/L HCl), and tested (diagnostic, 0.01 mol/L each of HCl, NaCl, and MSG) for proper function and stability. After successful testing, each water extract was analyzed five times. Instrument control, data acquisition, and data processing were performed with Alphasoft software (Alpha MOS., Toulouse, France). Taste screening analysis was used to rank the samples according to taste attributes on a 0 to 12 intensity scale. Principal component analysis (PCA) was performed to discriminate the response signals from the seven sensors. The heat map was created using a web-based interactive next-generation clustered heat map (NG-CHM) builder (https://build.ngchm.net/NGCHM-web-builder/) (accessed on 30 August 2022) with hierarchical clustering using the Euclidean distance metric with Ward’s agglomeration method.

## 4. Statistical Analysis

Statistical analysis was carried out using Statistica 13.0 (StatSoft, Kraków, Poland). A two-way ANOVA for flour type, percentage of substitution, and their interaction was used to test bread features at a significance level of *p* ≤ 0.05. When the Levene test indicated significant differences, a post-hoc Fisher’s least significant difference (LSD) test was performed. The results are presented as mean ± standard deviation. The primary data obtained in the consumer evaluation study were subjected to statistical calculations, and on their basis a regression analysis was performed for each of the tested insect meal variants. Multiple regression was used to quantify the relationships between multiple independent variables, such as appearance, smell, texture, and taste, and the dependent variable acceptance. Additionally, Dixon’s Q test was performed to eliminate gross errors. The value of Q was lower than the value determined from the Qcr tables each time, which means that the doubtful result was an element of the sample for α = 0.05.

## 5. Conclusions

Analysis of the obtained products showed that organoleptic acceptance was influenced by the type of edible insect flour used. This was a result of both the taste and smell sensations, as well as the texture of the tested product. Bars with the addition of buffalo worm flour were rated the highest, which contrasts with bars with the addition of mealworm flour. This evaluation could be based on the volatile compound profile, in particular the presence of compounds such as 3-methylobutanoic acid, hexanal, and 2,3-pentanedione in mealworm flour. Bars enriched with buffalo worm and cricket flours were high in butanal and furfural, which give products a chocolate and caramel taste and which may have contributed to the higher organoleptic evaluation of these bars. Regardless of the above, all bars containing insect flour showed an improvement in nutritional characteristics, especially in terms of protein quality, which was characterized by high biological value; leucine and lysine were identified as the limiting amino acids.

## Figures and Tables

**Table 1:** Chemical composition of raw materials and products (g/100 g).

Samples	Protein	Ash	Fat	Dietary Fiber			
				Insoluble Fraction	Including Chitin	Soluble Fraction	Total
BW	49.51 ^i^ ± 0.16	4.71 ^i^ ± 0.03	26.44 ^b^ ± 0.22	12.96 ^i^ ± 0.04	7.33 ^i^ ± 0.01	0.00 ^a^ ± 0.00	12.96 ^k^ ± 0.04
CF	55.18 ^j^ ± 0.11	4.34 ^h^ ± 0.07	29.01 ^d^ ± 0.04	18.48 ^j^ ± 0.06	9.92 ^h^ ± 0.01	0.24 ^b^ ± 0.01	18.72 ^l^ ± 0.05
TM	45.39 ^h^ ± 0.06	3.86 ^g^ ± 0.01	14.29 ^a^ ± 0.06	11.56 ^h^ ± 0.07	6.93 ^g^ ± 0.02	0.36 ^c^ ± 0.02	11.92 ^j^ ± 0.05
CN	21.50 ^g^ ± 0.03	2.53 ^f^ ± 0.00	45.62 ^i^ ± 0.48	5.19 ^d^ ± 0.01	0.00 ^a^ ± 0.00	0.67 ^e^ ± 0.06	5.86 ^e^ ± 0.05
HN	16.64 ^f^ ± 0.08	2.15 ^e^ ± 0.01	64.31 ^j^ ± 0.37	9.58 ^g^ ± 0.08	0.00 ^a^ ± 0.00	1.49 ^j^ ± 0.00	11.07 ^i^ ± 0.08
ST	10.78 ^a^ ± 0.02	1.49 ^a^ ± 0.01	36.20 ^h^ ± 0.06	3.14 ^a^ ± 0.03	0.00 ^a^ ± 0.00	0.84 ^g^ ± 0.01	3.98 ^a^ ± 0.02
BW15	13.23 ^b^ ± 0.15	1.51 ^a^ ± 0.02	34.07 ^f^ ± 0.01	4.14 ^b^ ± 0.06	0.77 ^b^ ± 0.01	0.85 ^g^ ± 0.04	4.99 ^b^ ± 0.09
BW30	14.06 ^d^ ± 0.01	1.57 ^c^ ± 0.01	32.62 ^e^ ± 0.16	5.20 ^d^ ± 0.04	1.91 ^e^ ± 0.01	0.84 ^g^ ± 0.03	6.04 ^f^ ± 0.07
CF15	13.48 ^c^ ± 0.04	1.56 ^b^ ± 0.01	32.91 ^e^ ± 0.08	4.88 ^c^ ± 0.07	1.01 ^c^ ± 0.01	0.75 ^f^ ± 0.04	5.63 ^d^ ± 0.03
CF30	15.51 ^e^ ± 0.04	1.73 ^d^ ± 0.01	26.69 ^c^ ± 0.11	6.04 ^f^ ± 0.01	2.35 ^f^ ± 0.01	0.53 ^d^ ± 0.01	6.57 ^h^ ± 0.03
TM15	13.11 ^b^ ± 0.12	1.52 ^a^ ± 0.01	35.48 ^g^ ± 0.23	4.14 ^b^ ± 0.09	0.77 ^b^ ± 0.01	1.04 ^i^ ± 0.01	5.18 ^c^ ± 0.11
TM30	13.69 ^c^ ± 0.16	1.57 ^c^ ± 0.00	29.35 ^d^ ± 0.23	5.46 ^e^ ± 0.03	1.53 ^d^ ± 0.01	0.95 ^h^ ± 0.02	6.41 ^g^ ± 0.05

Values in the same row marked with different letters are statistically significantly different at *p* < 0.05. BW—buffalo worm (*A. diaperinus*) flour, CF—cricket (*A. domesticus*) flour, TM—*T. molitor* flour (mealworm flour), ST—standard bar, BW15 and BW30—bars with 15% and 30% addition of buffalo worm flour, CF15 and CF30—bars with 15% and 30% addition of cricket flour, TM15 and TM30—bars with 15% and 30% addition of *T. molitor* flour, CN—cashew nut, HN—hazelnuts.

**Table 2:** Amino acid profile of raw materials and enriched products.

Samples	Protein (%)	Essential Amino Acids (mg/g of Protein)								Total EAA
		His	Ile	Leu	Lys	Met	Phe	Thr	Val	
BW	49.51 ^i^ ± 0.16	40.36 ^h^ ± 0.10	48.46 ^cb^ ± 1.13	74.78 ^b^ ± 1.96	73.17 ^hg^ ± 2.16	22.21 ^bc^ ± 2.68	48.76 ^b^ ± 1.87	44.98 ^c^ ± 1.02	64.20 ^cd^ ± 1.29	416.91 ^c^ ± 7.88
CF	55.18 ^j^ ± 0.11	25.85 ^c^ ± 0.17	44.19 ^b^ ± 0.26	79.14 ^b^ ± 0.58	57.26 ^c^ ± 0.65	19.70 ^a^ ± 0.85	37.11 ^a^ ± 0.31	41.04 ^b^ ± 0.43	62.40 ^c^ ± 0.76	366.93 ^b^ ± 2.37
TM	45.39 ^h^ ± 0.06	35.55 ^g^ ± 0.47	47.38 ^bc^ ± 0.78	77.47 ^b^ ± 1.28	60.85 ^dc^ ± 0.98	20.14 ^ab^ ± 2.19	36.87 ^a^ ± 0.56	43.89 ^c^ ± 0.85	67.62 ^de^ ± 1.15	389.79 ^b^ ± 4.06
CN	21.50 ^g^ ± 0.03	18.79 ^b^ ± 0.	32.35 ^a^ ± 0.89	57.83 ^a^ ± 1.45	39.55 ^b^ ± 0.86	23.70 ^cb^ ± 2.52	37.00 ^a^ ± 0.65	28.55 ^a^ ± 0.76	44.81 ^b^ ± 1.16	282.58 ^a^ ± 8.56
HN	16.64 ^f^ ± 0.08	21.63 ^a^ ± 0.66	31.37 ^a^ ± 1.17	57.23 ^a^ ± 1.93	24.39 ^a^ ± 0.75	26.30 ^d^ ± 1.37	37.44 ^a^ ± 1.29	26.58 ^a^ ± 0.76	40.13 ^a^ ± 1.38	265.09 ^a^ ± 7.36
ST	10.78 ^a^ ± 0.02	31.41 ^ef^ ± 3.00	63.46 ^g^ ± 6.00	109.67 ^e^ ± 10.55	68.82 ^fg^ ± 7.74	40.39 ^g^ ± 1.79	71.91 ^f^ ± 6.48	56.88 ^g^ ± 5.10	77.35 ^ih^ ± 6.96	519.88 ^e^ ± 44.12
BW15	13.23 ^b^ ± 0.15	30.12 ^e^ ± 0.33	56.43 ^f^ ± 0.36	94.71 ^dc^ ± 0.79	66.11 ^ef^ ± 2.61	36.99 ^e^ ± 1.05	61.73 ^de^ ± 0.51	50.78 ^ed^ ± 0.30	69.51 ^efg^ ± 0.31	466.39 ^d^ ± 3.84
BW30	14.06 ^d^ ± 0.01	33.21 ^fe^ ± 0.66	57.29 ^f^ ± 0.88	95.29 ^dc^ ± 1.59	69.46 ^gh^ ± 1.55	35.78 ^f^ ± 0.62	62.41 ^ed^ ± 2.51	52.93 ^fe^ ± 0.57	72.31 ^fgh^ ± 0.62	478.70 ^d^ ± 7.32
CF15	13.48 ^c^ ± 0.04	28.09 ^dc^ ± 0.41	55.80 ^ed^ ± 0.35	97.42 ^d^ ± 0.44	64.08 ^e^ ± 1.54	36.28 ^f^ ± 0.28	60.69 ^de^ ± 2.19	51.31 ^edf^ ± 0.26	70.97 ^efg^ ± 0.59	464.64 ^d^ ± 3.87
CF30	15.51 ^e^ ± 0.04	26.79 ^cd^ ± 0.36	52.70 ^d^ ± 1.09	91.13 ^c^ ± 1.66	60.05 ^dc^ ± 1.17	33.83 ^e^ ± 1.36	54.31 ^c^ ± 1.13	49.22 ^d^ ± 1.09	68.28 ^ef^ ± 1.50	436.32 ^c^ ± 7.87
TM15	13.11 ^b^ ± 0.12	30.89 ^ef^ ± 1.63	57.27 ^f^ ± 2.74	99.30 ^d^ ± 4.79	62.28 ^dce^ ± 4.49	34.62 ^ef^ ± 1.04	61.25 ^de^ ± 2.97	52.48 ^fe^ ± 2.59	73.19 ^gh^ ± 2.62	471.26 ^d^ ± 20.91
TM30	13.69 ^c^ ± 0.16	31.93 ^ef^ ± 2.29	57.30 ^f^ ± 3.667	98.40 ^d^ ± 6.46	66.51 ^ef^ ± 4.98	36.87 ^f^ ± 0.85	58.00 ^cd^ ± 3.61	54.03 ^f^ ± 2.86	74.25 ^hgf^ ± 4.39	477.29 ^d^ ± 28.55

Values in the same row marked with different letters are statistically significantly different at *p* < 0.05 ± SD. EAA—essential amino acids, HN—hazelnut, CN—cashews nuts, CF—cricket *A. domesticus*, TM—mealworm *T. molitor*, BW—buffalo worm *A. diaperinus*.

**Table 3:** Nutritional value of the protein of raw materials and enriched products.

Samples	AAS (%)							
	His	Ile	Leu	Lys	Thr	Val	AAA	SAA
BW	252.24	161.53	122.59	152.43	179.93	160.49	338.68	156.63
CF	161.59	147.31	129.74	119.29	164.16	156.60	233.49	126.13
TM	222.20	157.95	127.01	126.77	175.57	169.05	271.87	144.82
CN	117.43	107.83	94.81	82.40	114.18	112.01	158.532	165.31
HN	135.22	104.58	93.82	50.82	106.33	100.33	152.964	195.59
ST	196.31	211.52	179.78	143.37	227.52	193.37	275.83	317.37
BW15	188.23	188.09	155.27	137.74	203.13	173.78	270.24	272.26
BW30	207.57	190.96	156.22	144.72	211.72	180.78	296.59	265.28
CF15	175.55	186.01	159.71	133.51	205.25	177.42	260.84	264.90
CF30	167.48	175.67	149.39	125.10	196.88	170.71	244.28	253.41
TM15	193.03	190.89	162.79	129.75	209.90	182.97	272.44	262.53
TM30	199.58	190.99	161.31	138.56	216.11	185.63	280.26	269.59

AAS—amino acid score, HN—hazelnut, CN—cashews nuts, CF—cricket *A. domesticus*, TM—mealworm *T. molitor*, BW—buffalo worm *A. diaperinus*.

**Table 4:** Fatty acid profile of nut bars’ raw materials and enriched products (g/100 g).

Fatty Acids	BW	CF	TM	CN	HN	ST	BW15	BW30	CF15	CF30	TM15	TM30
C16:0	27.21 ^b^ ± 0.02	28.90 ^a^ ± 0.00	23.34 ^c^ ± 0.14	9.14 ^f^ ± 0.01	5.99 ^l^ ± 0.01	8.76 ^g^ ± 0.00	9.61 ^e^ ± 0.02	11.64 ^d^ ± 0.00	7.39 ^k^ ± 0.01	7.63 ^j^ ± 0.01	7.81 ^i^ ± 0.02	8.20 ^h^ ± 0.02
C18:0	8.55 ^b^ ± 0.01	11.85 ^a^ ± 0.04	4.68 ^j^ ± 0.04	8.25 ^c^ ± 0.04	3.06 ^k^ ± 0.03	5.60 ^f^ ± 0.01	5.72 ^e^ ± 0.00	5.03 ^i^ ± 0.01	5.63 ^f^ ± 0.00	5.32 ^h^ ± 0.00	5.97 ^d^ ± 0.01	5.43 ^g^ ± 0.01
C18:1 cis oleic	34.12 ^i^ ± 0.00	25.85 ^j^ ± 0.00	54.87 ^h^ ± 0.36	62.97 ^g^ ± 0.03	82.44 ^a^ ± 0.06	70.96 ^d^ ± 0.05	70.51 ^e^ ± 0.08	69.56 ^f^ ± 0.02	73.88 ^b^ ± 0.00	73.87 ^b^ ± 0.00	73.10 ^c^ ± 0.06	73.11 ^c^ ± 0.00
C18:2 (9.12) n-6 cis	24.84 ^a^ ± 0.02	23.36 ^b^ ± 0.05	7.80 ^l^ ± 0.04	17.70 ^c^ ± 0.01	7.87 ^k^ ± 0.00	12.75 ^d^ ± 0.02	12.35 ^e^ ± 0.01	10.93 ^j^ ± 0.00	11.73 ^g^ ± 0.001	11.81 ^f^ ± 0.00	11.06 ^h^ ± 0.02	11.00 ^i^ ± 0.02
C18:2 (9.12) n-6 trans	0.10 ^b^ ± 0.00	0.28 ^a^ ± 0.00	0.11 ^b^ ± 0.01	n.d.	n.d.	0.02 ^d^ ± 0.00	0.02 ^cd^ ± 0.00	0.03 ^c^ ± 0.00	0.01 ^d^ ± 0.00	0.02 ^cd^ ± 0.00	0.01 ^d^ ± 0.00	0.01 ^d^ ± 0.00
∑SFA	37.76 ^b^ ± 0.01	40.75 ^a^ ± 0.08	28.02 ^c^ ± 0.28	17.39 ^d^ ± 0.01	9.05 ^j^ ± 0.04	14.36 ^g^ ± 0.01	15.32 ^f^ ± 0.04	16.67 ^e^ ± 0.02	13.02 ^i^ ± 0.01	12.95 ^i^ ± 0.01	13.78 ^h^ ± 0.04	13.630 ^h^ ± 0.015
∑MUFA	34.12 ^i^ ± 0.02	25.852 ^j^ ± 0.036	54.871 ^h^ ± 0.314	62.97 ^g^ ± 0.02	82.44 ^a^ ± 0.06	70.96 ^d^ ± 0.03	70.51 ^e^ ± 0.06	69.56 ^f^ ± 0.02	73.88 ^b^ ± 0.01	73.86 ^b^ ± 0.00	73.10 ^c^ ± 0.06	73.11 ^c^ ± 0.00
∑PUFA	24.94 ^a^ ± 0.03	23.65 ^b^ ± 0.04	7.91 ^j^ ± 0.03	17.70 ^c^ ± 0.01	7.87 ^k^ ± 0.02	12.76 ^d^ ± 0.02	12.37 ^e^ ± 0.02	10.94 ^i^ ± 0.00	11.74 ^g^ ± 0.00	11.83 ^f^ ± 0.00	11.08 ^h^ ± 0.02	11.09 ^h^ ± 0.0124
∑PUFA/∑SFA	0.69 ^h^ ± 0.00	0.58 ^j^ ± 0.00	0.28 ^k^ ± 0.00	1.02 ^a^ ± 0.00	0.87 ^c^ ± 0.00	0.89 ^d^ ± 0.00	0.81 ^e^ ± 0.00	0.66 ^i^ ± 0.00	0.90 ^c^ ± 0.00	0.91 ^b^ ± 0.00	0.80 ^g^ ± 0.00	0.81 ^f^ ± 0.00

Values in the same row marked with different letters are statistically significantly different at *p* < 0.05 ± SD. HN—hazelnut, CN—cashews nuts, CF—cricket *A. domesticus*, TM—mealworm *T. molitor*, BW—buffalo worm *A. diaperinus*.

**Table 5:** Consumer acceptance of bars.

Samples	Descriptors					
	Appearance	Color	Smell	Texture	Smell	Overall Acceptance
ST	6.56 ^b^ ± 0.82	6.40 ^b^ ± 1.00	6.40 ^cb^ ± 1.04	6.32 ^ba^ ± 1.03	6.48 ^dc^ ± 0.65	6.48 ^cb^ ± 0.77
BW15	6.37 ^b^ ± 1.01	6.22 ^b^ ± 1.09	6.00 ^bc^ ± 1.71	6.41 ^ba^ ± 0.93	6.30 ^cd^ ± 0.95	6.26 ^bc^ ± 0.81
BW30	5.92 ^b^ ± 1.61	5.76 ^ba^ ± 1.56	5.60 ^ba^ ± 1.35	5.88 ^a^ ± 1.36	6.28 ^cd^ ± 0.94	6.28 ^bc^ ± 0.89
CF15	6.23 ^b^ ± 1.18	6.04 ^b^ ± 1.37	5.31 ^ba^ ± 1.44	5.58 ^a^ ± 1.53	5.58 ^cb^ ± 1.42	5.73 ^ba^ ± 1.22
CF30	5.10 ^a^ ± 1.78	5.28 ^a^ ± 1.81	4.90 ^a^ ± 1.74	5.66 ^a^ ± 1.34	5.76 ^cb^ ± 1.41	5.79 ^ba^ ± 1.35
TM15	6.32 ^b^ ± 0.84	6.32 ^b^ ± 0.88	5.21 ^ba^ ± 1.41	5.91 ^a^ ± 1.29	5.35 ^b^ ± 1.76	5.76 ^ba^ ± 1.44
TM30	6.50 ^b^ ± 0.76	6.46 ^b^ ± 0.76	5.23 ^ba^ ± 1.66	5.73 ^a^ ± 1.34	4.54 ^a^ ± 1.98	4.77 ^a^ ± 1.84

Values in the same column marked with different letters are statistically significantly different at *p* < 0.05 ± SD. ST—standard bar, BW15 and BW30—bars with 15% and 30% addition of buffalo worm flour, CF15 and CF30—bars with 15% and 30% addition of cricket flour, TM15 and TM30—bars with 15% and 30% addition of *T. molitor* flour.

## Data Availability

Not applicable.

## Supplementary Materials

The following supporting information can be downloaded at: https://www.mdpi.com/article/10.3390/molecules27238472/s1, Figure S1: Protein profiles of the selected raw materials analyzed by SDS-PAGE; Figure S2: Nut bars with various levels of edible insects flour substitution—Overall appearance; Table S1: Amino acid profile of raw materials and products; Table S2 Amino acid profile of raw materials and products; Table S3: Concentration of individual volatile compounds in edible insect flours (g/100 g).

## Sample Availability

Samples of the compounds are available from the authors.
