# Supplementary material for: Chemical Composition, Nutritional Value, and Acceptance of Nut Bars with the Addition of Edible Insect Powder
Source: Molecules. 2022 Dec 2;27(23):8472. doi: 10.3390/molecules27238472 (PMC9741150; doi:10.3390/molecules27238472)
Supplement: Supplementary file 1 [file molecules-27-08472-s001.zip › molecules-2023814-supplementary.pdf]

Table S1 Examples of commercially available products containing edible insects (adapted from www.bugburger.se)

	Name	Picture
1	Bar (protein bar energy bar)	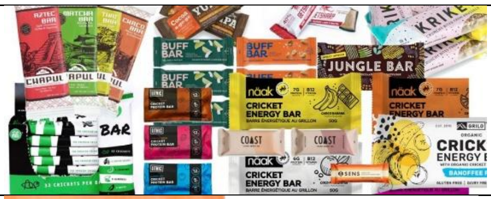
2	Beer	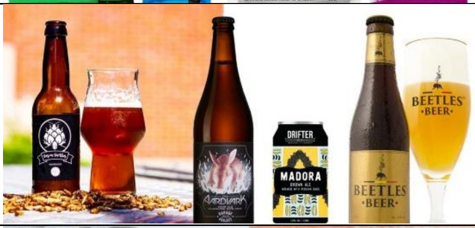
3	Bread	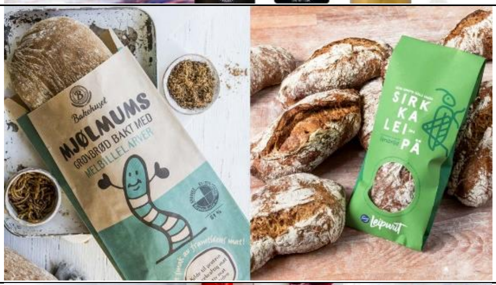
4	Burgers	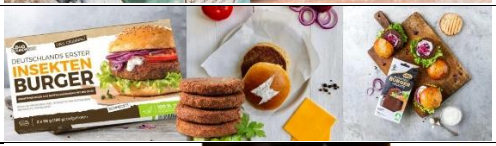
5	Cookies	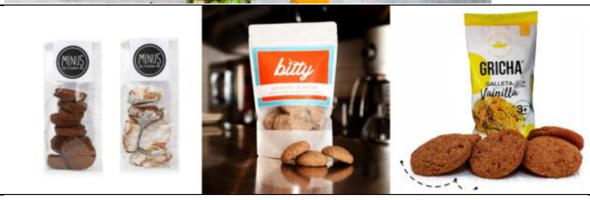
6	Crackers	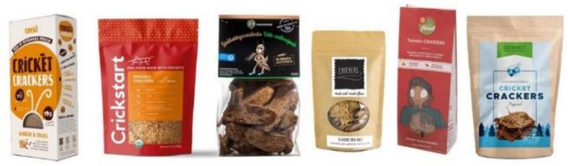
7	Crisps/Chips	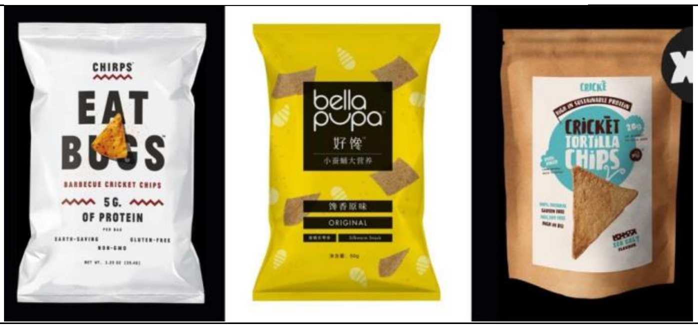

<table>
<tr><td>8</td><td>Noodles</td><td>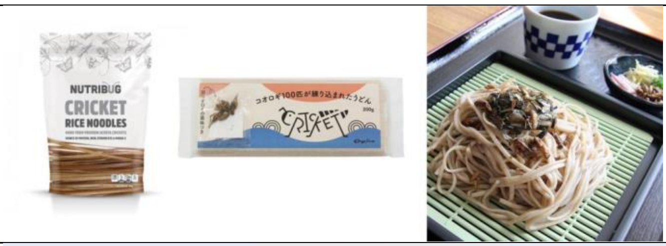
</td></tr>
<tr><td>9</td><td>Oil</td><td>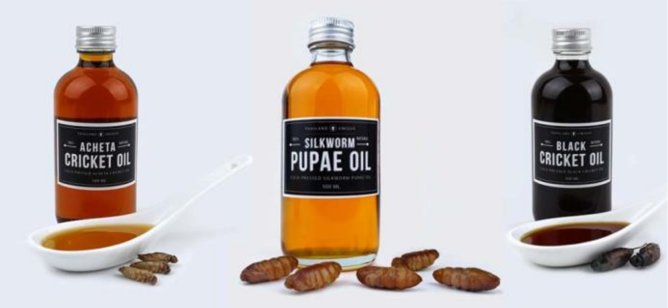
</td></tr>
<tr><td>10</td><td>Pancake mix</td><td>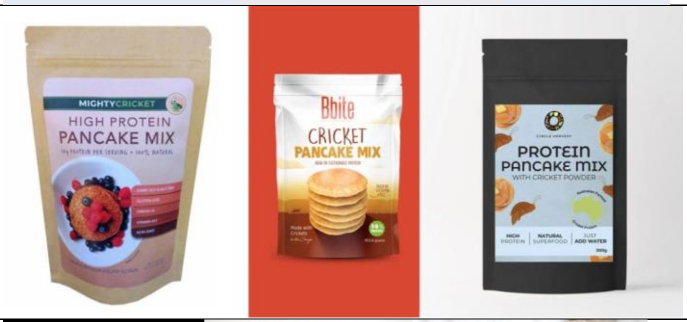
</td></tr>
<tr><td>11</td><td>Pasta</td><td>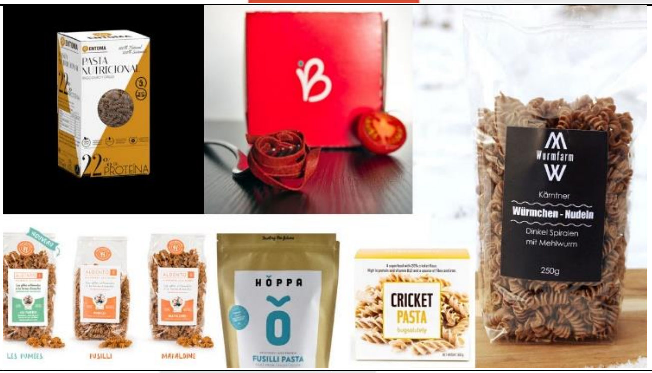
</td></tr>
<tr><td>12</td><td>Protein powder/shakes</td><td>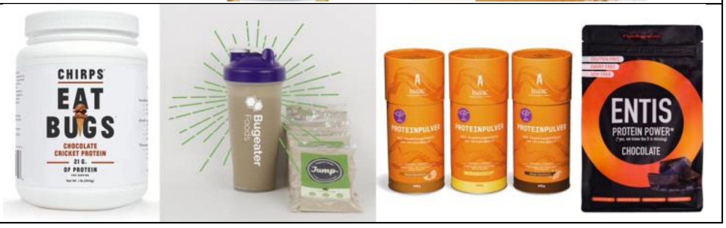
</td></tr>
</table>

Table S2 Amino acid profile of raw materials and products.

	Non-essential amino acids (non-EAA) $mg \cdot g^{-1}$ of protein									Total non-EAA
	Alanine	Arginine	Aspartic acid	Cysteine	Glutamic acid	Glycine	Proline	Serine	Tyrosine	
BW	$75.85^{d}\pm1.25$	$62.03^{a}\pm1.70$	$97.81^{cb}\pm2.34$	$11.40^{a}\pm0.36$	$144.18^{b}\pm3.42$	$51.24^{fe}\pm1.10$	$74.34^{de}\pm1.50$	$47.50^{b}\pm1.05$	$90.10^{h}\pm2.44$	$654.45^{b}\pm14.39$
CF	$99.77^{f}\pm1.43$	$70.21^{bc}\pm0.71$	$94.56^{bc}\pm1.23$	$10.71^{a}\pm0.60$	$126.30^{a}\pm1.69$	$60.26^{h}\pm0.70$	$64.27^{b}\pm0.88$	$48.43^{b}\pm0.77$	$58.62^{f}\pm0.74$	$633.15^{b}\pm7.56$
TM	$80.32^{e}\pm1.51$	$60.36^{a}\pm1.15$	$91.06^{b}\pm1.52$	$10.98^{a}\pm0.41$	$130.72^{a}\pm2.35$	$56.72^{g}\pm1.04$	$79.56^{f}\pm1.32$	$49.673^{b}\pm0.97$	$74.59^{g}\pm1.6$	$633.98^{b}\pm11.09$
CN	$32.14^{a}\pm0.64$	$91.15^{d}\pm2.11$	$76.70^{a}\pm2.39$	$16.809^{b}\pm2.03$	$182.88^{c}\pm3.19$	$35.57^{a}\pm0.72$	$28.10^{a}\pm0.95$	$42.15^{a}\pm0.82$	$27.99^{a}\pm0.44$	$533.48^{s}\pm12.82$
HN	$40.48^{b}\pm1.43$	$124.77^{e}\pm3.29$	$91.97^{bc}\pm2.45$	$15.75^{b}\pm0.65$	$209.27^{de}\pm5.04$	$40.58^{b}\pm1.47$	$27.56^{a}\pm0.99$	$39.95^{a}\pm1.10$	$25.27^{a}\pm0.90$	$615.59^{b}\pm16.23$
ST	$65.93^{c}\pm4.97$	$72.59^{cb}\pm6.16$	$114.68^{e}\pm10.34$	$30.43^{e}\pm0.63$	$277.31^{g}\pm23.60$	$45.97^{dc}\pm4.00$	$80.34^{f}\pm7.08$	$93.52^{f}\pm8.20$	$41.18^{b}\pm2.95$	$821.95^{e}\pm66.36$
BW15	$64.95^{c}\pm0.26$	$66.29^{b}\pm0.68$	$106.04^{d}\pm0.43$	$25.27^{d}\pm0.32$	$222.36^{ef}\pm1.64$	$44.02^{c}\pm0.67$	$70.20^{cd}\pm1.67$	$77.50^{de}\pm0.47$	$49.07^{de}\pm1.05$	$725.70^{dc}\pm6.08$
BW30	$71.72^{d}\pm0.49$	$69.05^{bc}\pm1.19$	$110.60^{d}\pm1.03$	$24.54^{d}\pm0.04$	$211.03^{def}\pm1.60$	$47.90^{de}\pm0.52$	$72.043^{e}\pm0.64$	$75.71^{d}\pm0.69$	$59.19^{f}\pm1.99$	$741.79^{d}\pm6.85$
CF15	$73.79^{d}\pm0.42$	$69.56^{bc}\pm0.39$	$105.68^{d}\pm1.02$	$25.23^{d}\pm0.26$	$223.15^{ef}\pm0.95$	$47.96^{de}\pm0.21$	$72.06^{de}\pm0.51$	$78.41^{de}\pm0.44$	$46.26^{cd}\pm2.03$	$742.10^{d}\pm2.07$
CF30	$73.79^{d}\pm5.15$	$68.31^{bc}\pm1.47$	$104.82^{d}\pm2.09$	$23.12^{c}\pm0.88$	$195.04^{c}\pm4.41$	$48.67^{def}\pm1.08$	$65.97^{bc}\pm1.74$	$72.14^{cd}\pm1.24$	$45.84^{c}\pm1.85$	$697.70^{c}\pm17.44$
TM15	$68.43^{cd}\pm3.60$	$69.24^{bc}\pm3.05$	$106.55^{d}\pm5.03$	$24.67^{d}\pm0.38$	$230.88^{fe}\pm11.18$	$47.89^{de}\pm2.44$	$75.73^{ef}\pm4.98$	$81.14^{ed}\pm4.16$	$50.453^{ed}\pm3.07$	$754.98^{d}\pm35.82$
TM30	$73.92^{d}\pm4.69$	$70.28^{bc}\pm4.24$	$110.07^{d}\pm5.08$	$25.64^{d}\pm0.45$	$207.96^{d}\pm12.08$	$50.75^{ef}\pm3.17$	$74.36^{de}\pm5.06$	$78.98^{de}\pm4.25$	$56.90^{f}\pm3.67$	$748.87^{d}\pm42.02$

HN – hazelnut, CN – cashews nuts, CF - cricket *A. domesticus*, TM - mealworm *T. molitor*, BW - buffalo worm *A. diaperinu*

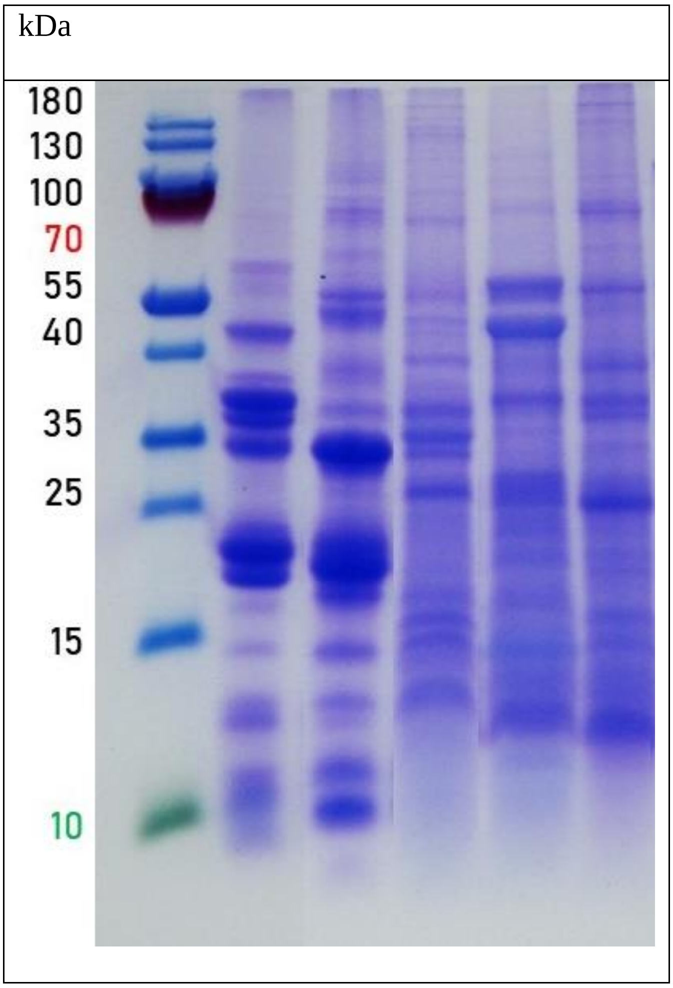

HN CN CF TM BW

Figure S1 Protein profiles of selected raw materials analyzed by SDS-PAGE

Explanatory notes:

HN – hazelnut, CN – cashews nuts, CF - cricket *A. domesticus*, TM - mealworm *T. molitor*, BW - buffalo worm *A. diaperinus*

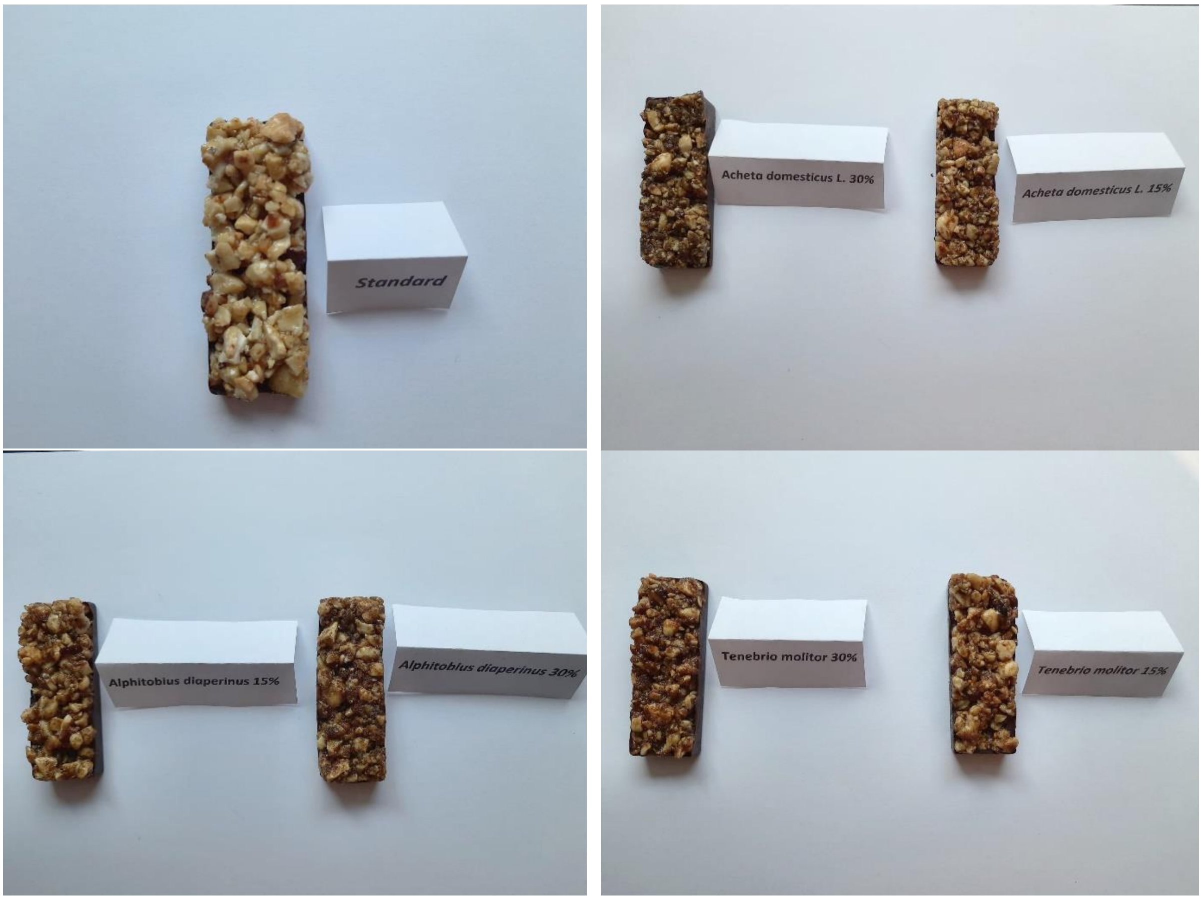

Figure S2 Nut bars with various levels of edible insects flour substitution - overall appearance

Table S3 Concentration of individual volatile compounds in edible insect flours (g/100g).

Volatile compounds	BW	CF	TM
Acetaldehyde	$4.50^{b}\pm0.14$*	$4.56^{c}\pm0.13$	$0.08^{a}\pm0.02$
2-Propanol	$26.04^{c}\pm0.18$	$21.70^{b}\pm0.17$	$7.79^{a}\pm0.11$
2-Methylpropanal	$0.01^{a}\pm0.01$	$1.83^{c}\pm0.05$	$0.05^{b}\pm0.02$
Butanal	$0.67^{a}\pm0.04$	$2.34^{c}\pm0.08$	$1.55^{b}\pm0.05$
Butan-2-one	$7.36^{c}\pm0.09$	$0.28^{a}\pm0.04$	$2.64^{b}\pm0.08$
Ethyl Acetate	$0.29^{b}\pm0.03$	$5.61^{c}\pm0.09$	$0.15^{a}\pm0.03$
Acetic acid	$7.40^{c}\pm0.11$	$1.14^{b}\pm0.07$	$0.64^{a}\pm0.06$
3-Methylbutanal	$1.87^{b}\pm0.08$	$1.28^{a}\pm0.11$	$1.92^{c}\pm0.13$
2,3-Pentanedione	$2.42^{b}\pm0.07$	$1.80^{a}\pm0.09$	$14.82^{c}\pm0.19$
Hexanal	$5.10^{a}\pm0.10$	$11.75^{c}\pm0.18$	$8.70^{b}\pm0.21$
Butanoic acid	$9.06^{c}\pm0.12$	$4.79^{b}\pm0.15$	$0.18^{a}\pm0.06$
Furfural	$3.51^{c}\pm0.11$	$0.37^{a}\pm0.07$	$0.54^{b}\pm0.08$
3-Methylbutanoic acid	$11.61^{a}\pm0.24$	$35.31^{b}\pm0.69$	$55.16^{c}\pm0.87$
1-Hexanol	$0.88^{c}\pm0.09$	$0.38^{a}\pm0.05$	$0.67^{b}\pm0.07$
Heptanal	$3.13^{c}\pm0.10$	$0.98^{b}\pm0.06$	$0.73^{a}\pm0.08$
Ethyl hexanoate	$2.40^{c}\pm0.11$	$0.52^{b}\pm0.04$	$0.02^{a}\pm0.01$
2,5-Dimethylpyrazine	$0.74^{c}\pm0.06$	$0.01^{a}\pm0.01$	$0.52^{b}\pm0.06$
2,3-Dimethylpyrazine	$0.82^{a}\pm0.07$	$0.91^{b}\pm0.09$	$0.82^{a}\pm0.07$
2,4-Heptadienal	$0.50^{b}\pm0.04$	$1.25^{c}\pm0.12$	$0.39^{a}\pm0.05$
Benzaldehyde	$3.05^{c}\pm0.09$	$0.24^{a}\pm0.08$	$0.35^{b}\pm0.04$
Hexanoic acid	$0.19^{b}\pm0.09$	$0.44^{c}\pm0.09$	$0.07^{a}\pm0.02$
Trimethylpyrazine	$0.09^{b}\pm0.02$	$0.03^{a}\pm0.02$	$0.69^{c}\pm0.03$
Tetramethylpyrazine	$0.09^{a}\pm0.03$	$0.84^{c}\pm0.10$	$0.82^{b}\pm0.06$
2-Phenylethanol	$1.53^{c}\pm0.10$	$0.16^{b}\pm0.08$	$0.08^{c}\pm0.02$
Phenylethyl acetate	$0.01^{a}\pm0.01$	$0.01^{a}\pm0.01$	$0.01^{a}\pm0.01$
Maltol	$0.26^{c}\pm0.04$	$0.07^{b}\pm0.01$	$0.02^{a}\pm0.01$
gamma-Nonalactone	$0.66^{c}\pm0.07$	$0.51^{b}\pm0.08$	$0.23^{a}\pm0.08$
Eugenol	$1.34^{b}\pm0.10$	$0.12^{a}\pm0.03$	$0.12^{a}\pm0.04$
delta-Nonalacton	$0.01^{a}\pm0.01$	$0.09^{b}\pm0.02$	$0.01^{a}\pm0.01$
Pentyl octanoate	$1.95^{b}\pm0.12$	$0.01^{a}\pm0.01$	$0.01^{a}\pm0.01$
Myristicin	$0.65^{c}\pm0.06$	$0.20^{b}\pm0.13$	$0.01^{a}\pm0.01$
E-nerolidol	$0.35^{c}\pm0.05$	$0.12^{b}\pm0.13$	$0.01^{a}\pm0.01$

*Values in the same row marked with different letters are statistically significantly different at $p < 0.05$ ± SD.
CF - cricket *A. domesticus*, TM - mealworm *T. molitor*, BW - buffalo worm *A. diaperinus*
